# Achieving and Maintaining Safety in Healthcare Requires Unwavering Institutional and Individual Commitments

**DOI:** 10.7759/cureus.13192

**Published:** 2021-02-07

**Authors:** Daniele Rigamonti, Karen H Rigamonti

**Affiliations:** 1 Neurological Surgery, Johns Hopkins Medicine International, Johns Hopkins Health System Corporation, Baltimore, USA; 2 Medicine, Johns Hopkins Medicine International, Johns Hopkins Health System Corporation, Baltimore, USA

**Keywords:** safety culture climate, healthcare, safety, harm, medical errors, adverse events, rca, coaching, near misses, close calls

## Abstract

In 2000, “To Err Is Human” brought to light the fact that the estimated number of people dying from medical errors occurring in hospitals exceeded those that die from motor vehicle accidents (MVAs), breast cancer, or acquired immunodeficiency syndrome (AIDS) - three causes receiving far more public attention. The report prompted the gradual adoption of safety processes developed in the nuclear and aviation industries.

However, sophisticated engineering solutions to operations are not enough. High and low mortality hospitals have similar processes and procedures, but low-mortality hospitals are more proficient at recognizing and managing serious complications as they unfold. This ability to rescue a deteriorating situation (resilience) reflects a healthier safety culture. Organizations move within the safety space in the direction of either more or less resilience depending on the fluctuation of their safety culture.

Improving resilience requires transforming learned safety practices into a “habit” in conjunction with accepting accountability. Personal accountability means commitment to safe practices along with effective and transparent reporting of near misses/close calls and adverse events (AEs). Institutional accountability means putting safety first by ensuring the availability of appropriate resources, role leadership modeling, and effective management of sentinel events (SEs) to reduce harm occurrence and re-occurrence. This requires a more robust root cause analysis (RCA) process to guarantee that action plans produce strong and effective corrective measures.

Synergistic coaching interventions include instilling the awareness that failure can and will happen, mapping team talents, and assessing gaps. These interventions will optimize group expertise, reaffirming the concept of institutional and personal accountability. The unending performance of drills will sustain the group resilience under both expected and unexpected conditions.

Given the strong correlation between practice environment and outcomes, sustained improvement of the safety climate will produce more robust safety behaviors and ultimately better outcomes.

## Introduction and background

In 2000, the publication of “To Err Is Human” by the Institute of Medicine brought to the public attention the fact that the estimated number of people dying in any given year from medical errors occurring in hospitals exceeded those that die from motor vehicle accidents (MVAs), breast cancer, or acquired immunodeficiency syndrome (AIDS) - three causes then receiving far more public attention [1]. The document also highlighted the complexity of the problem that Is compounded by many factors that include the increasing complexity of providing medical care and the decentralized and fragmented nature of the health care delivery system without clear lines of accountability. It is easier for something to go wrong, when patients see multiple providers in different settings, often with no access to complete information, than when care is better coordinated. Later studies further corroborated this disheartening state of affairs. McGlynn et al. found that half of the Americans fail to get the effective treatment they need [2]. Chassin et al. determined that a third of Americans receive treatments of little or no benefit [3]. Leape et al. confirmed that 10% or more patients are significantly harmed by preventable mishaps [4]. In order to systematically reduce harm in clinical medicine, healthcare organizations needed to ensure a new framework for analyzing risk [5], as well as a culture of error recognition, honesty, accountability, and fair settlements of injuries [6]. A comprehensive approach to improving patient safety is needed, because this complex problem requires thoughtful, multifaceted responses. The purpose of this review is to underpin unwavering institutional and individual commitments to improve patients’ safety.

## Review

The comparison of the risk/harm occurring in healthcare to the risk/harm occurring in other dangerous industries, such as the aviation and nuclear industries, led to the examination and adoption of safety processes, such as checklists, that these High-Reliability Organizations (HROs) developed from the analysis of their accidents. Paradoxically, however, in spite of significant improvements using checklists (i.e., the decreased incidence of catheter-related bloodstream infections), they produce unintended consequences: “nothing threatens safety so much as the complacency induced when an organization thinks that the problem is solved” [7]. 

Studies of the outcomes linked to the implementation of more mature processes and procedures further corroborated the suspicion that better and more sophisticated engineering of the operations would not necessarily reduce the harm caused by medical errors [8]. Another element plays a critical role: rescue. “This is what distinguished the great from the mediocre. They did not fail less. They rescued more” [9].

High-mortality hospitals do not do a worse job of controlling risk or preventing post-surgical complications than low-mortality hospitals. High-mortality hospitals are less resilient when dealing with an unexpected event, are less able to absorb the strain, bounce back, and recover. They, in other words, “fail to rescue” an imminent catastrophe, while low-mortality hospitals are more proficient at recognizing and managing serious complications as they unfold [8]. The pressure of the deteriorating situation makes rescuing very challenging because it exposes weaknesses in relationships among team members stemming from expectation of task performance, trustworthiness, and self-respect. Eliminating weaknesses in relationships is crucial to improve the team’s grasp of what is evolving and enhance its ability to rescue the situation/increase resilience [10-12].

HROs manage to increase resilience by sharpening alertness and awareness of unfolding conditions through the implementation of five interrelated behavioral processes together creating a state of Collective Mindfulness [13]. These processes are Preoccupation with Failure, Reluctance to Simplify Interpretations, Sensitivity to Operations, Commitment to Resilience, and Deference to Expertise. 

The implementation of these behavioral processes in healthcare has proven effective in decreasing medication errors [14,15], lowering nurses’ emotional exhaustion [16], decreasing unit-level turnover in areas with high rates of adverse advents [16], and ultimately lowering mortality rates [14,17]. In addition, high levels of trust in managers and use of care pathways seem to amplify the above benefits [18]. 

It is not surprising that, at this juncture, most healthcare organizations have adopted highly effective interventions to decrease hospital-acquired infections and increase medication safety. Data, however, show that safety culture varies across hospitals and within each hospital depending on a variety of factors. While administration/management generally perceive the safety culture to be high [19], nurses, particularly those at the bedside, perceive practice environment to be worse compared to managers and other healthcare workers. These perceived differences between management personnel, nurses, and other caregivers are a consistent finding in the literature. Compared to physicians, nurses reported significantly worse working conditions, perceptions of management, and job satisfaction [20]. Operating room (OR) nurses, who were given the highest overall ratings of teamwork, rated teamwork of surgeons the lowest [21]. Surgeons had the lowest overall ratings of teamwork, followed by anesthesiologists, despite the fact that surgeons and anesthesiologists rated teamwork within their own disciplines the highest [21]. Furthermore, only a minority of OR nurses felt that conflicts are appropriately resolved, compared to the majority of anesthesiologists and surgeons [22]. A survey of non-surgical intensive care units (ICUs) produced similar results: only one-third of nurses rated the quality of collaboration and communication with physicians as high or very high, in contrast to two-thirds of physicians rating high or very high the collaboration with nurses [23].

A professional nurse practice environment has been linked to better quality of care and patient safety outcomes. Hospitals known for good nursing care, "magnet" hospitals, have decentralized decision making to the level of the nursing unit, strong nursing leadership, recognition of professional nurse autonomy, adequate staffing, flexible scheduling, accountability, and nursing responsibility for quality patient care. They also have significantly lower Medicare patient mortality rates compared to matched controls [24]. In Magnet hospitals, moreover, nurses experience better relationships with physicians. These organizational characteristics became incorporated into the Nursing Work Index Revised (NWIR) and a subsequent instrument called the Practice Environment Scale of the Nursing Work Index (PES-NWI) [24]. Favorable practice environment scores on the PES-NWI are related to higher nurse-reported quality of care and better surgical outcomes for cancer patients [25]. In particular, the PES-NWI subscale Collegial Nurse-Physician Relations has been found to be an important predictor of nurse-reported quality of care [26]. Higher scores on Collegial Nurse-Physician Relations subscale have also been associated with lower 30-day mortality after controlling for patient and hospital factors [27]. 

Current data, however, suggest that in spite of years of hard work, twenty years after the publication of “To Err is Human”, the harm produced during the delivery of healthcare remains a major concern. Healthcare is still not safe [28]. This state of affairs has many explanations: “we do not get safety behind us”….”organizations... that organize... repeatedly and continually are likely to achieve greater safety and reliability than those organizations that don’t… (because of) the binding safety cultures they create through the enactment of these processes and associated activities” [29].

While the safety policies and procedures that we are using to train staff are sound and evidence-based, organizations over time “move in the safety space” in the direction of either more or less resilience [30]. Developing and maintaining a safety culture requires actions that focus on safety-relevant practices [31] and relentless training in order to make them become a “habit” [32]. Truthfully, to avoid the occurrence and recurrence of adverse events (AEs) requires an unwavering institutional and individual commitment. 

As troublesome as it may seem, healthcare organizations rarely investigate and take action before a serious injury occurs. This situation could be corrected by paying stronger attention to close calls that occurring 300 times more often than SEs are more likely to point to latent conditions, rather than individual acts, that foretell error [33]. Leaders, therefore, need to strengthen reporting near misses/close calls [34]. Studying these events, the team will understand what could go wrong, identify the early signs of a situation with the potential of turning sour [33,35], learn the necessary recovery strategies, and avoid future reoccurrences. Another system deficiency often resides in an inadequate root cause analysis (RCA) process that simplifies the interpretation of a SE losing sight of the big picture and failing to discover the gaps in the system. Furthermore, the RCA process frequently recommends action plans (re-education, writing a policy) that are weak and less likely to prevent event recurrence [36]. A strong RCA action plan, furthermore, requires a commitment to follow-up on the outcome of any recommendation [37]. Together these actions will increase the “Reluctance to simplify interpretations” and the “Sensitivity to operations” helping to understand and eventually eliminate the gap between what actually occurred in the workplace and what people expected to occur.

Improving safety also means “Commitment to resilience” i.e., reinforcing all the processes that produce collective mindfulness within the multidisciplinary team [12]. The training should focus on specific activities aimed at strengthening resilience [29,38] in order to make them become a “habit” [32]. Leaders need strong and sustainable solutions effective at preventing the occurrence and recurrence of AEs. Leaders need to remember that safety is about character: “Are we trustworthy? Can our patients be sure that we are doing everything we know to make health care safe, by ourselves and our colleagues? Are we honest, open, and forthcoming with our patients and ourselves when things go wrong?” [39]. Accountability starts with institutions. Institutional accountability means putting safety first, ensuring appropriate resources, and leadership role modeling. Senior managers’ words and deeds receive enormous attention and greatly influence how frontline workers and middle managers perceive what their organization values and rewards [11,39,40]. Leaders need to develop managers that people want to follow, create a learning organization, and find opportunities within risks [41,42]. Inspiring leaders help institutions to adopt policies and practices that demonstrate “Deference to expertise” by encouraging equal attention to advice, regardless of the source. Information quality, rather than the attributes of the advice giver, helps obtain diverse expert perspectives and ensure collaborative medical decision making, promoting better and more effective patient care [43].

Coaching the frontline members of the team to instill the awareness that failure can and will happen (“Preoccupation with Failure”) reinforces the criticality of each individual in maintaining the safety of the complex system in which they live and work [10,40]. Coaching the surgeons and the anesthesiologists, for example, is critical because the OR is inhabited by hierarchical, mixed-gender clinical teams that are prone to conflict ranging from constructive differences of opinion to discord and distraction that ultimately jeopardize patient safety [44]. Although coaching is perceived as challenging by physicians, especially surgeons, due to social and peer expectations related to identity, the safe space of intentional coaching allows participants to practice vulnerability without the pressures of professional norms [3]. Coaching that helps mapping team talents/skills and recognizing gaps optimizes group behavior, exercising “Commitment to resilience” and “Deference to expertise”. 

Another important element in the development and maintenance of a safety culture is the reaffirmation of personal accountability [45]. It behooves the healthcare organizations to find the right balance between blaming mistakes on systems and holding individual providers accountable for their everyday behaviors. These behaviors include actively participating in safety practices (hands washing, preop antibiotics, protocols/checklists, reconciliation, time-out, etc.), reporting errors and AEs, identifying hazards and taking action. A safe and just culture recognizes that the failure to follow clear rules in the face of well-functioning systems is a violation; repeated violations in health care, as in any industry, should have consequences [45] reaffirming that while there will be no blaming for errors, there will be no tolerance for misconduct [39]. 

Lastly, assessing group resilience (“the expression of how people, alone or together, cope with everyday situations, large and small, by adjusting their performance to the condition, under both expected and unexpected conditions”) [46] should be a recurrent activity. “System safety is an illusory concept (past performance cannot predict the future safety” [47]. Safety is a dynamic non-event: safety is preserved by timely human adjustments [47]. Performing regular drills will improve the team’s ability at monitoring, anticipating, responding, and learning [46].

The coordinated implementation of team coaching, enhanced reporting of AEs (close calls and SEs), strengthened RCA process and action plans, heightened accountability and enhanced teamwork will produce an improvement in the safety climate as measured by validated instruments [48,49]. It is known that a correlation exists between prevailing culture and safety behaviors and outcomes [50]. Although definitive proof can only come from high-quality trials, we believe that improving safety climate in any organization will begin to have beneficial effects on the rate of medication errors, on the level of nurses’ emotional exhaustion and turnover in units with high rates of adverse advents, and ultimately on morbidity and mortality rates.

## Conclusions

In spite of many years of hard work, twenty years after the publication of “To Err is Human”, harm produced during the delivery of healthcare remains a major concern. Healthcare is still not safe. While most healthcare organizations have adopted sound interventions to decrease the occurrence of adverse events, the impact of these interventions varies because of uneven practice and implementation. The literature consistently points to perceived safety culture differences between management and frontline staff, and between nurses and other caregivers. Poor teamwork and inadequate conflict resolution play a significant role. Safety requires an unending commitment to creating binding cultures by enacting these processes and associated activities. To augment the resilience of a team, a unit, or an organization, it is necessary to improve their grasp of what is evolving by enhancing teamwork through coaching. Coaching will strengthen the social infrastructure enabling staff to speak up, map skills, and defer to expertise, while assigning institutional and individual accountability. Coaching will also facilitate the difficult process of learning from errors and performing regular drills to maintain the acquired skills.
